# Correction to “Impact of Donor Age, Sex, and ABO Compatibility on Outcomes After Matched Unrelated Donor Hematopoietic Stem Cell Transplantation for Acute Leukemia: A Retrospective Single‐Center Cohort Study”

**DOI:** 10.1002/hsr2.72949

**Published:** 2026-08-04

G. Kheiri, M. Mashayekhi, S. Saeii, et al., “Impact of Donor Age, Sex, and ABO Compatibility on Outcomes After Matched Unrelated Donor Hematopoietic Stem Cell Transplantation for Acute Leukemia: A Retrospective Single‐Center Cohort Study,” *Health Science Reports* 9 (2026): e72775. https://doi.org/10.1002/hsr2.72775.

The name of the (eighth author) was misspelled and has been updated from “Saeid Mohammadi” to “Saeed Mohammadi.”

The online version of this article has been corrected accordingly.

We apologize for this error.

